# Environmentally friendly splints for limb immobilisation: a systematic review

**DOI:** 10.1308/rcsann.2024.0037

**Published:** 2024-05-24

**Authors:** JA Mawhinney, SJM Parker, A Selby, NA Johnson

**Affiliations:** Pulvertaft Hand Centre, Royal Derby Hospital, University Hospitals of Derby and Burton NHS Foundation Trust, UK

**Keywords:** environmentally friendly, biodegradable, ecological, Woodcast, splints, limb

## Abstract

**Introduction:**

Climate change is estimated to be the biggest global health threat of the 21^st^ century, and has prompted calls to move away from processes in healthcare associated with high energy consumption and greenhouse gas emission. In musculoskeletal medicine, splints are widely used for limb immobilisation. These have typically been made from single-use materials such as gypsum, although in recent years purportedly environmentally friendly splints have been designed. In this systematic review, we set out to assess the clinical effectiveness of all commercially available environmentally friendly splinting materials, including Woodcast^®^.

**Methods:**

The AMED (Allied and Complementary Medicine Database), CINAHL^®^ (Cumulative Index to Nursing and Allied Health Literature), Cochrane Central Register of Controlled Trials, Embase^®^, Emcare^®^ and MEDLINE^®^ databases were searched to identify studies assessing the clinical effectiveness of biodegradable and environmentally friendly splints prior to paper review and data extraction. Formal quantitative synthesis was not possible owing to the substantial heterogeneity in the study designs and outcome measures.

**Results:**

Six papers met the inclusion criteria, all investigating one particular splint material (Woodcast^®^). One was a case series, two were cohort studies and three were randomised controlled trials. Primary outcome measures were heterogeneous but the environmentally friendly splints were generally equivalent to traditional splint materials. Studies were mostly at a high risk of bias.

**Conclusions:**

There is limited research assessing ‘green’ splints in practice although the data suggest similarity with existing materials and no substantial safety concerns. Further scrutiny of the clinical effectiveness and environmental credentials of such splints is also required.

## Introduction

Climate change is considered to be the biggest global health threat of the 21^st^ century, resulting from unbridled energy consumption and greenhouse gas emissions.^1^ In response to this, the National Health Service (NHS) in the UK launched the Greener NHS campaign in 2020, with a target of reaching net zero for carbon emissions that the NHS controls directly by 2040 and for those that the NHS has the ability to influence by 2045.^2^ It is acknowledged that this will require substantial commitment from the entire workforce to find new and innovative ways of delivering healthcare in more sustainable ways. NHS England specifically identifies medicines and medical devices as a major source of carbon emissions and as such, one of the greatest opportunities for change.^2^ Clinicians are encouraged to deliver improved patient outcomes with a reduced impact on the environment through more efficient use of supplies, low-carbon substitutions and product innovation.

In musculoskeletal medicine, one such medical device is the splint, commonly used for extremity immobilisation and aiding rehabilitation.^3,4^ The most widely used splinting material is plaster of Paris (POP).^5^ The principal agent in POP is gypsum, which has been used for limb immobilisation for over 170 years, with only the addition of binding agents and stabilisers as a means of modernisation. Its longevity reflects numerus advantages including high conformity, low pressure areas and shear stresses, high porosity and low cost whereas its disadvantages include weight, durability, radio-opacity and cumbersome application. Furthermore, despite gypsum itself being a naturally occurring compound, POP casts are slow to degrade and difficult to recycle as well as requiring substantial water consumption during production.^6^

Alternatives to POP include polyurethane, fibreglass and plastic polymer-based splints.^7^ However, several authors have questioned the safety and appropriateness of using such materials, which contain toxic and potentially harmful chemicals known to cause skin irritation and asthma-related conditions in the user.^8–10^ Moreover, it must be considered whether the use of non-biodegradable and single-use splints, in contradiction to the Greener NHS ideals,^2^ can be justified.

In order to reconcile good quality patient care and an environmentally conscious approach to health delivery, a new generation of splinting material has been developed. The most prominent of these is the Woodcast^®^ (Dassiet [formerly Onbone], Espoo, Finland). The manufacturers describe a novel non-toxic, composite material made from clean aspen wood chips and a biodegradable polymer that can be composted after use. This reduces its contribution to landfill and offers a smaller carbon footprint than alternatives.^11^ Personal communication from the manufacturers of Woodcast^®^ suggests this takes approximately six months to a year to biodegrade in soil (or less time in compost).

For application, the material is cut to shape and heated without water, which then allows 3D moulding about the patient. It sets within three minutes (or faster with active cooling). The non-toxic and hypoallergenic properties mean that synthetic gloves need not be worn, further reducing the use of consumables. Once set, the material is waterproof and washable. If additional adjustments are required as treatment progresses, the same splint can be heated and remoulded as necessary, reducing waste and costs associated with creating an entirely new splint.

Lab-based mechanical studies, principally by the manufacturer, comparing Woodcast^®^ with other commonly used synthetic splinting materials appear to show equivalence or better results in properties of functional stiffness, ring stiffness, bending and compression (simulated heel strike).^12–15^ Nevertheless, given the potential variability in application and clinical usage, and the importance of industry-independent research, uneasiness remains as to whether these results translate to achieving reliable equivalent clinical outcomes for patients. In this systematic review, we set out to assess the clinical effectiveness of all commercially available environmentally friendly splinting materials, including the Woodcast^®^.

## Methods

The protocol for this systematic review was registered in the PROSPERO database (CRD42023372219). The University of York’s Centre for Reviews and Dissemination guidance for undertaking reviews in healthcare was followed,^16^ and reporting was performed in accordance with the 2020 update to the PRISMA (Preferred Reporting Items for Systematic reviews and Meta-Analyses) statement for developing study protocols and reporting systematic reviews.^17^ All data are available on request.

### Literature search

The following databases were searched by an experienced clinical librarian: AMED (Allied and Complementary Medicine Database), CINAHL^®^ (Cumulative Index to Nursing and Allied Health Literature), Cochrane Central Register of Controlled Trials, Embase^®^, Emcare^®^ and MEDLINE^®^. A ‘grey literature’ search incorporating any available information outside the usual scientific registries, including industry reports, was also performed. A multi-layered search strategy was developed utilising the terms cast, splint, brace and immobilisation alongside biodegradable, environment, compost, toxic, wood and recycle (as well as permutations thereof). Woodcast^®^ was used as a separate search term given previous knowledge in the area. The manufacturer of the Woodcast^®^ was also approached to identify any publications the company was aware of. Any additional articles identified (including from the references of relevant papers) were added. All papers from start of registry records to 24 February 2023 were included in the search.

### Inclusion and exclusion criteria

Studies were eligible for inclusion in this systematic review if they were published, in any language, and featured the use of any self-determined environmentally friendly or biodegradable splint for limb immobilisation in both adult and paediatric populations while assessing a clearly stated outcome pertaining to its clinical effectiveness or patient satisfaction. Prospective, retrospective, observational and interventional studies were all eligible for inclusion. Studies were excluded if they were performed outside a direct clinical context. Search results were added to a shared online database for independent blinded assessment (Rayyan).^18^ Following removal of duplicate records, two reviewers (JM and SP) assessed the abstract of each paper in turn. Conflicts were resolved through a meeting held in person. The full text of papers was then reviewed in a similar fashion. Any further disagreements during the review process were resolved by formal group discussion involving all team members.

### Data extraction and risk of bias assessment

Data were extracted by two reviewers (JM and SP) and entered into a shared database. Collected data included paper title, study author and digital object identifier, country of study team, study design, number of patients, inclusion criteria, age, sex, type of splint, duration of splint, primary outcome, secondary outcomes and any complications.

The methodological quality of all papers was assessed using the Critical Appraisal Skills Programme’s standardised critical appraisal checklists.^19^ Randomised studies were additionally appraised by two reviewers (JM and AS) using the revised Cochrane RoB 2 tool,^20^ with the risk of bias for the primary outcome measure of each study reported. All studies were reviewed separately by both reviewers before an in-person discussion, where the final score was allocated. Again, any disagreements were resolved by formal group discussion with the wider team.

### Statistical analysis

Formal quantitative synthesis was not possible owing to the substantial heterogeneity in the study designs and outcome measures.

## Results

**Figure 1:**
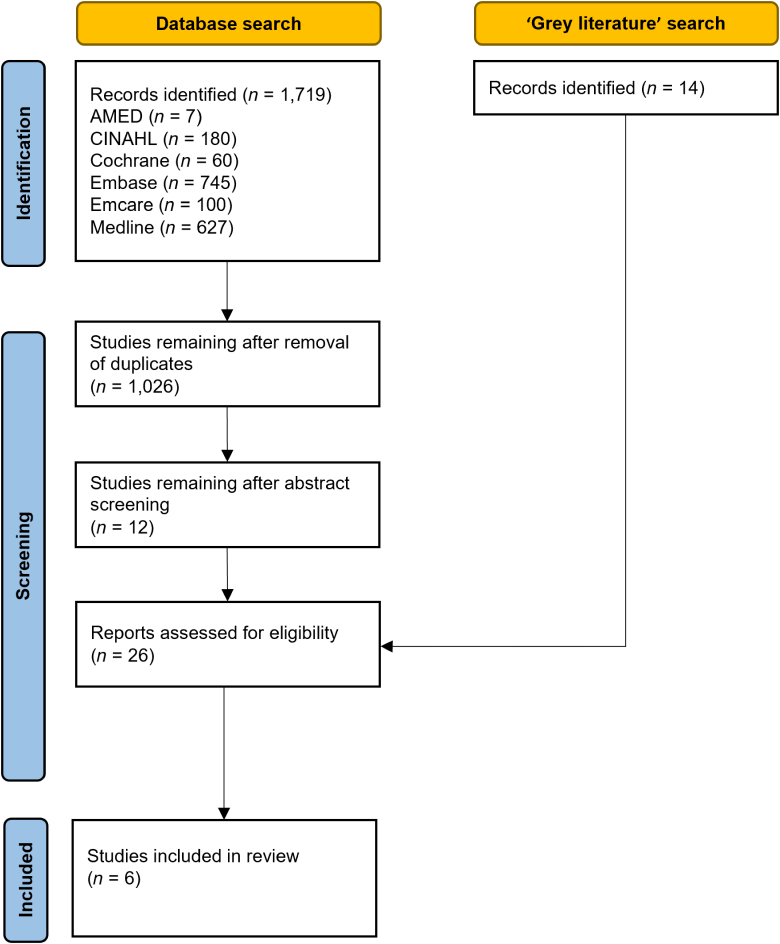
Flowchart of study selection

Of the 1,719 records identified through the initial database search and the 14 additional ‘grey literature’ records, 6 papers met the inclusion criteria (Figure 1).^12,15,21–24^ These studies are summarised in Table 1. One additional study investigating Woodcast^®^ was identified on a clinical trials register although this trial was not complete and was therefore was not included.

**Table 1:** Summary of the six included studies

Study	Country	Study design	Number of patients	Cast type	Splinting duration
Lindfors, 2012^21^	Finland	Case series	33	Short arm	4 weeks
Hirsimäki, 2014^22^	Finland	Cohort	30	Ankle	4 weeks (following 2 weeks with an alternative splint)
Lindfors, 2014^12^	Finland	Cohort	67	Scaphoid	4 weeks
Martynov, 2020^15^	Germany	RCT	170	Short arm	21 days (≤7 years), 28 days (≥8 years)
Singh, 2020^23^	India	RCT	50	Ponseti	Splinting duration was the outcome measure
Gwilym, 2020^24^	UK	RCT	120	Short arm	5 weeks
RCT = randomised controlled trial					

One paper was designated as a case series and two as cohort studies. These were produced from the same group in Finland.^12,21,22^ The other three studies were randomised controlled trials.^15,23,24^ Four of the studies were performed for upper limb immobilisation^12,15,21,24^ and two for lower limb.^22,23^ Owing to the heterogeneity in study outcomes, a formal meta-analysis was not conducted.

Table 2 displays the outcome measures and major results of the included studies. No primary outcome measure was identified in the three non-randomised studies. Among the randomised studies, the primary outcome measures were identified as observer-identified splint breakage,^15^ ability to achieve full correction in patients with congenital talipes equinovarus deformity^23^ and patient-rated wrist evaluation (PRWE) score.^24^ In the study where PRWE was used, it was also separated into the pain and function subscales. Overall, the Woodcast^®^ splint was similar to the control splint in each of these studies. However, a formal sample size calculation was not performed in two and in the third, only 25% of the predicted patients were recruited in the context of a feasibility study.

**Table 2:** Outcome measures and results for the six included studies

Study	Outcome measures	Comparator groups	Results	Adverse events
Lindfors, 2012^21^	Primary outcome not specified • Application time • Applicator-reported ease of use • Patient-reported comfort • Description of complications • Mechanical properties (non-clinical)	• Woodcast^®^ not otherwise specified	• 5.3 mins application time • 31/33 applicators preferred a composite cast • Patient satisfaction reported as “high”	• 10/27 patients reported splint pressure, 10 reported rubbing and 2 required repositioning. 1 patient had loss of fracture position but this was not attributed to the splint.
Hirsimäki, 2014^22^	Primary outcome not specified • Application time • Applicator-reported ease of use • Patient-reported comfort • Description of complications	• Woodcast^®^ 2mm soft, Woodcast^®^ 2mm and Woodcast^®^ 4mm (removable semi-rigid orthosis) • Woodcast^®^ 2mm (non-removable rigid cast)	• 25.5 mins application time (group 1) vs 15.2 mins (group 2) • Patient satisfaction reported as “high”	• Group 1: 0/13 complications • Group 2: 12/17 complications, including 6 cases of superficial maceration and 3 of focal compression
Lindfors, 2014^12^	Primary outcome not specified • Application time • Applicator-reported ease of use • Patient-reported comfort • Cast changes • Description of complications • Mechanical stiffness (non-clinical)	• Woodcast^®^ 2mm and Woodcast^®^ ribbon • Woodcast^®^ 2mm soft and Woodcast^®^ ribbon	• 15/18 mins application time (non-removal/removal respectively; group 1) vs 14/18 mins (group 2) • 63/66 applicators preferred a composite cast • 15/26 patients reported as comfortable (group 1) vs 20/30 (group 2) • 5 casts changed (group 1) vs 2 (group 2)	• Group 1: 5/26 patients complained of compression or discomfort and 5 of odour • Group 2: 4/30 patients complained of compression or discomfort and 7 of odour
Martynov, 2020^15^	• Stress stability of the splint material (splint breakage; primary outcome) • Parental and patient satisfaction • Medical staff satisfaction • Other splint-related outcomes	• Woodcast^®^ not otherwise specified • Dynacast^®^	• 3.6% breakage (Woodcast^®^) vs 3.5% (control; NS)	• 25.0% adverse events in Woodcast^®^ group vs 27.9% in Dynacast^®^ group (*p*=0.720)
Singh, 2020^23^	• Efficacy of correction (number of splints required to achieve correction; primary outcome) • Cast-related problems • Cast application time • Cast removal time	• Woodcast^®^ 2mm • POP	• 4.87 casts required to achieve correction (Woodcast^®^) vs 4.35 (control; NS)	• 10/111 complications in Woodcast^®^ group (relating to cast slippage, tight cast, softening or cracking, skin abrasions, skin redness) • 12/95 minor complications in POP group
Gwilym, 2020^24^	• PRWE (primary outcome) • Complications and safety concerns • Fracture displacement • Patient adherence • Health-related quality of life (EQ-5D-5L)	• Woodcast^®^ not otherwise specified • Fibreglass	• Mean PRWE total score 30 (Woodcast^®^) vs 29 (control; not statistically tested)	• No serious adverse events • 7 minor complications in Woodcast^®^ group (relating to straps falling off, skin irritation and pain) vs 1 in fibreglass group (skin irritation)
NS = not significant; POP = plaster of Paris; PRWE = patient-rated wrist evaluation				

Secondary outcomes described included application time, and subjective applicator and patient satisfaction with the splint material. Satisfaction tended to be described less thoroughly in the earlier studies but was summarised as a five-point scale across comfort, weight, temperature, smell and perceived level of support. Splints were generally similar across secondary outcome measures.

### Risk of bias

Table 3 summarises the risk of bias assessment for the studies. Two of the randomised controlled trials were found to have a high risk of bias, largely due to a lack of blinding in patients and researchers, and concerns regarding the measurement of the outcomes. Two studies reported financial disclosures related to the manufacturer of Woodcast^®^^12,24^ although the two earliest studies were also produced by the same team as a group that later declared a conflict of interest.^21,22^

**Table 3:** Risk of bias assessment for the six included studies

Study	RoB 2 score^20^	Financial and conflicts-of-interest disclosure
Lindfors, 2012^21^	N/A	None declared
Hirsimäki, 2014^22^	N/A	None declared
Lindfors, 2014^12^	N/A	Both authors act as clinical advisors for the company manufacturing Woodcast^®^
Martynov, 2020^15^	High	None declared
Singh, 2020^23^	Low	None declared
Gwilym, 2020^24^	High	Unrestricted research grant from the company manufacturing Woodcast^®^

## Discussion

The use of more environmentally friendly splints will be an important transition in musculoskeletal medicine and is aligned with the Greener NHS long-term agenda.^2^ Clinicians are slowly moving away from traditional POP-based immobilisation, with ongoing investigation into alternative regimes,^25^ and this therefore offers an important opportunity to push forwards with more environmentally friendly alternatives. The present study identifies early use of a splint material described by the manufacturer as more ecologically favourable although there is not enough evidence currently to confirm whether this is clinically comparable with pre-existing splint materials.

### Outcome measures

The outcome measures described were heterogenous across the six studies included in the systematic review, making cumulative analysis difficult. The most important outcome measure in such studies should be non-inferiority in terms of direct clinical outcomes. Only one of the studies used a patient-rated outcome measure.^24^ Instead, there was a tendency to utilise non-clinical measures such as ‘time to make’; although this is important, it should clearly come second to direct clinical outcome measures.

Furthermore, there is also concern over the lack of a designated primary outcome in the earlier studies and absence of sample size calculations. Only one of the studies described a formal sample size calculation and even then, only a portion of the total predicted number of participants were recruited, with the trial acting as a feasibility study for future research.^24^

### Blinding

One of the major risks of bias identified across the studies was a lack of blinding in both patients and researchers. Evidently, it may be difficult to ensure blinding of patients, particularly when comparing with materials such as POP. Despite this, where possible, assessors should be blinded, such as in the study assessing congenital talipes equinovarus.^23^ The lack of blinding makes the use of patient-rated outcome measures challenging as participants can be influenced by which arm of the study they are in. For this reason, supplementing these with assessor-determined outcomes such as radiologically deemed fracture position or unity may be advised.

### Environmental credentials

A full assessment of the environmental credentials of these splints was not the research question of this review but this is clearly an important consideration. None of the included studies considered how the splints were disposed of in each setting. Personal communication from the manufacturers of Woodcast^®^ suggests a biodegradation time of six months to a year in soil and less in compost but this necessitates appropriate conditions (including heat, moisture and bacteria). There is also the possibility that the splints were simply incinerated, thereby negating any positive biodegradability effects. Moreover, it is important to consider whether there is simultaneous use of other, non-biodegradable splint materials in practice. For example, splints are often used in conjunction with liners or are trimmed with additional material in order to improve comfort. The studies included in this review did not consider these important factors.

Other potential positive environmental effects of such splints include using less water than traditional POP, being reusable and being able to be remodelled on multiple occasions.^21^ However, similar to above, these points have yet to be determined conclusively by independent assessment. More evidence is needed before this as well as the impact of the lifecycle and supply chain of environmentally friendly splint materials can be confirmed.

Finally, although it was also not part of this review, future research should investigate the financial implications of biodegradable splints and consider performing a full health economic assessment.

### Conflicts of interest

A further potential source of bias relates to the impact of industry on the production of research. Of the six papers included in this study, four were found to have been funded or supported in some way by the device manufacturer. Although industry-funded papers are important, such research must be interpreted in the context within which it is produced. For that reason, extra scrutiny is required, particularly in such studies where conflicts of interests are not readily disclosed.

### Limitations

One of the major limitations of this systematic review is that only one type of splint was investigated in the seven studies eligible for inclusion. While there are other reportedly biodegradable splints on the market, these have not yet undergone the same level of investigation as Woodcast^®^ and no studies investigating this were able to be included in this systematic review.

Nevertheless, despite the lack of evidence to support Woodcast^®^ (or alternatives) against existing splint material, there is some cause for optimism. No particular safety concerns were identified in this systematic review, with Woodcast^®^ being generally comparable with regular splints in this regard. Furthermore, preclinical research in industry-sponsored reports claims to demonstrate similar splint strength and faster manufacture times than alternatives.^26,27^ Finally, the increase in the number of studies investigating this particular topic is evidence itself of the increasing enthusiasm for moving towards environmentally friendly alternatives in healthcare and the eagerness of industry to innovate in this particular field.

## Conclusions

The wider use of environmentally friendly splints will be an important way in which musculoskeletal surgery can contribute to the Greener NHS initiative.^2^ However, there is a limited amount of research assessing the efficacy of such splints in clinical practice. Initial work seems promising but higher-quality evidence is needed before the equivalence or otherwise of these newer devices to pre-existing forms of splint can be confirmed. In addition, greater scrutiny over the environmental credentials of medical devices is required in order to ensure that these splints are more sustainable than older alternatives.
